# Changes in Fall Risk After Total Hip Arthroplasty for Dysplastic Hip Osteoarthritis

**DOI:** 10.1111/os.70296

**Published:** 2026-03-20

**Authors:** Takamune Asamoto, Yusuke Osawa, Yasuhiko Takegami, Hiroto Funahashi, Yuto Ozawa, Shiro Imagama

**Affiliations:** ^1^ Department of Orthopaedic Surgery Nagoya University Graduate School of Medicine Nagoya Japan

**Keywords:** assessment of fall risk, dysplastic hip osteoarthritis, fall, fall risk index, hip osteoarthritis, total hip arthroplasty

## Abstract

**Objective:**

Total hip arthroplasty (THA) improves function in patients with dysplastic hip osteoarthritis (DHOA). However, its effect on reducing fall risk remains unclear. This study aimed to evaluate fall risk following THA in patients with DHOA.

**Methods:**

This retrospective cohort study included 85 patients who had DHOA and underwent THA between September 2019 and September 2022 and were evaluated as having a preoperative fall risk (Fall Risk Index 5 items [FRI‐5] ≥ 6). They were categorized into two groups according to the FRI‐5 score 1 year postoperatively. Evaluation parameters included FRI‐5 score, age, sex, body mass index (BMI), Harris hip score (HHS), perceived leg length discrepancy (P‐LLD), and radiographic parameters. Logistic regression was used to assess risk factors for postoperative falls.

**Results:**

The FRI‐5 score significantly decreased from 7.79 (6.0–13.0) preoperatively to 4.56 (0–13.0) postoperatively (*p* < 0.001). The number of falls during the year decreased from 36 (42.4%) to 18 (21.2%) after surgery (*p =* 0.005). The high‐risk and low‐risk groups comprised 33 and 52 individuals, respectively. The high‐risk group was significantly older than the low‐risk group (*p* = 0.006). Postoperative P‐LLD was significantly large in the high‐risk group compared to that in the low‐risk group (*p* = 0.005). Preoperative and postoperative sagittal vertical axes (SVA) were significantly larger and preoperative lumbar lordosis (LL) was significantly lower in the high‐risk group than in the low‐risk group (*p* = 0.039, *p* = 0.034, and *p* = 0.021, respectively). Logistic regression analysis identified age (OR: 1.2, 95% CI: 1.05–1.36, *p* = 0.006), preoperative low LL (OR: 0.944, 95% CI: 0.892–0.999, *p* = 0.046), and postoperative P‐LLD (OR: 5.81, 95% CI: 1.23–27.5, *p* = 0.026) as significant factors associated with fall risk.

**Conclusion:**

THA for patients who have DHOA at high risk of falls reduces the likelihood for falls. Therefore, surgeons should plan surgeries considering the risk factors post‐THA.

## Graphical abstract

Total hip arthroplasty reduced fall risk in patients with dysplastic hip osteoarthritis at high fall risk. Older age, low preoperative lumbar lordosis, and postoperative perceived leg length discrepancy were associated with higher postoperative fall risk.

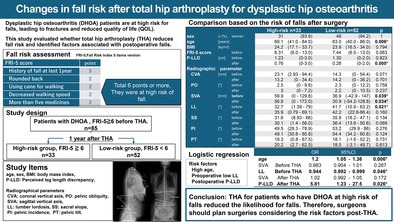

## 1 Introduction

The World Health Organization has identified falls as the second leading cause of unintentional injury–related deaths worldwide, making them a considerable concern in aging societies [1]. Hip osteoarthritis (HOA) is associated with a high risk of falls, with a reported fall rate of 45% among patients with HOA [2]. Total hip arthroplasty (THA) is considered the gold‐standard treatment for HOA and substantially improves hip function; however, its impact on fall risk remains unclear. Although THA may be expected to reduce fall risk by ameliorating pain and improving gait mechanics, previous studies have not consistently demonstrated a reduction in postoperative falls in patients with HOA.

Postoperative falls can result in severe complications, including dislocation and periprosthetic fractures [3, 4]. Prior reports suggesting increased fall risk after THA often did not assess preoperative fall risk and frequently included patients at inherently low risk [5, 6, 7]. More recent registry‐based analyses and meta‐analyses have shown mixed outcomes, with some reporting no increase in postoperative falls and others indicating a reduction in fall‐related injuries following improved mobility after THA [8]. Therefore, the effect of THA on fall risk, particularly among patients with high preoperative fall risk, requires further clarification.

Common risk factors for falls in older adults include aging, forward trunk inclination, and diminished muscle strength [9]. In patients with HOA, pain, gait abnormalities, and impaired balance are additional contributors to fall risk [10]. Dysplastic hip osteoarthritis (DHOA), which accounts for a substantial proportion of HOA cases in Japan, is associated with leg‐length discrepancy and coronal imbalance, potentially increasing fall risk [11]. Conversely, postoperative gait abnormalities have also been reported as risk factors for falls after THA [12].

Spinopelvic alignment influences fall risk in older adults [9], and THA has been shown to induce postoperative changes in spinopelvic alignment [13]. These findings suggest that both hip‐related and spinal–pelvic parameters may be important determinants of postoperative fall risk.

Given this background, we hypothesized that THA would reduce fall risk in patients with DHOA who have high preoperative fall risk, and that postoperative fall risk may be associated with patient demographics, hip function, and spinopelvic alignment. Therefore, we addressed the following clinical questions: (1) Does THA improve fall risk postoperatively? (2) Are patient demographic characteristics associated with post‐THA fall risk? (3) Is hip function associated with post‐THA fall risk? and (4) Does spinopelvic alignment contribute to post‐THA fall risk?

## 2 Material and Methods

### 2.1 Study Participants

This retrospective observational study adhered to the Strengthening the Reporting of Observational Studies in Epidemiology (STROBE) guidelines and was approved by our Institutional Review Board (2020‐0272).

All participants provided written informed consent prior to inclusion. This retrospective observational study included consecutive patients who underwent primary total hip arthroplasty (THA) at our institution between September 2019 and September 2022 and who completed a preoperative questionnaire assessment.

### 2.2 Inclusion and Exclusion Criteria

The study population consisted of patients with dysplastic hip osteoarthritis (DHOA) who underwent primary THA. The primary variable of interest was preoperative fall risk, which was assessed using a standardized questionnaire, and patients were classified according to their preoperative fall risk status.

A total of 259 consecutive patients were initially screened. Patients were excluded if they had femoral head osteonecrosis (*n* = 42), primary hip osteoarthritis (*n* = 33), traumatic hip osteoarthritis (*n* = 15), prior hip surgery including osteotomy (*n* = 6), rapidly destructive coxarthrosis (*n* = 5), or subchondral insufficiency fracture (*n* = 5). In addition, 65 patients with low preoperative fall risk were excluded. After applying these criteria, 85 patients with DHOA and high preoperative fall risk were included in the final analysis. The sex distribution was 5 men and 80 women, with a mean age of 65.4 (40.0–86.0) years and body mass index (BMI) of 24.0 (16.5–34.0) kg/m^2^ (Table 1).

**TABLE 1:** Patient characteristics.

			*n* = 85	
Age (years)	Median (range)		65.4	(40.0–86.0)
BMI (kg/m^2^)			24.0	(16.5–34.0)
Other side before THA	*n* (%)	Normal	45	(52.9)
		HOA	31	(36.5)
		THA	9	(10.6)
Other side after THA	*n* (%)	Normal	45	(52.9)
		HOA	17	(20.0)
		THA	23	(27.1)
Bi‐lateral THA	*n* (%)		14	(16.5)
HHS		Before THA	57.2	(31.0–85.0)
		After THA	88.6	(65.0–100)
Crowe classification	*n* (%)	I	67	(78.8)
		II	13	(15.6)
		III	4	(4.7)
		IV	1	(1.2)

Abbreviations: BMI, body mass index; HHS, Harris hip score; HOA, hip osteoarthritis; JHEQ, Japanese Orthopedic Association Hip‐Disease Evaluation Questionnaire; THA, total hip arthroplasty.

### 2.3 Fall Risk Assessment

We assessed fall risk using the Fall Risk Index 5 items version (FRI‐5) [14]. This assessment included patient self‐reports of the following factors over the past year: history of falls (5 points), cane use (2 points), rounded back (2 points), decreased walking speed (2 points), and intake of five or more medications (2 points). Patients with an FRI‐5 score of 6 or higher were considered at risk for falls. The questionnaire was administered the day before and 1 year after surgery. Changes in the FRI‐5 score before and after surgery, and the incidence of patients who experienced a fall during the year were investigated and compared. Subsequently, the patients were divided into two groups based on their fall risk at 1 year post‐surgery: those at high risk of falling (high‐risk group) and those at low risk of falling (low‐risk group).

### 2.4 Clinical Evaluation

The clinical variables analyzed included age, BMI, Harris Hip Score (HHS), perceived leg length discrepancy (P‐LLD), condition of the contralateral hip, and whether bilateral hip arthroplasty had been performed. HHS and P‐LLD were evaluated preoperatively and at 1 year postoperatively according to the method described by Koga et al. [15], and both variables were recorded as absolute values.

**FIGURE 1:**
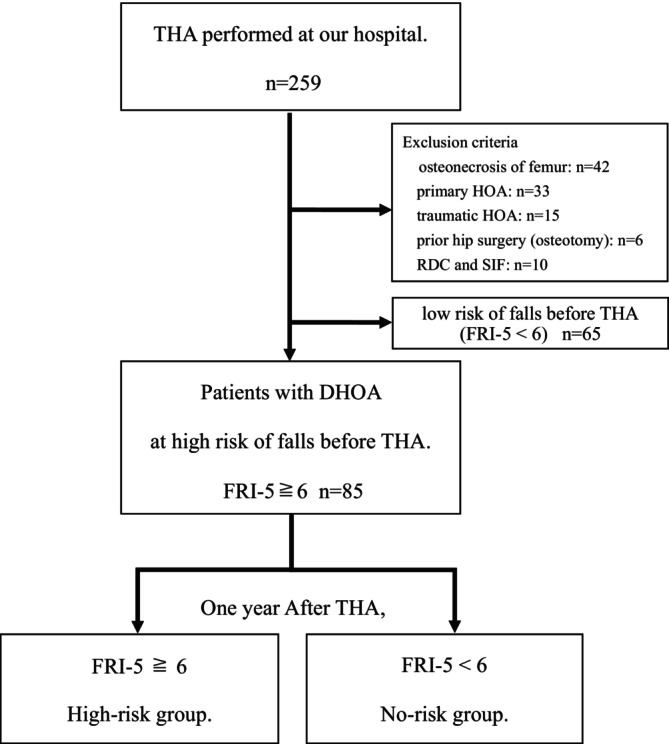
Study design. DHOA, dysplastic hip osteoarthritis; FRI‐5, fall risk index 5 items version; THA, total hip arthroplasty.

To assess P‐LLD, patients stepped barefoot onto a wooden spacer calibrated at 5‐mm intervals, and the height at which they perceived their leg lengths to be equal was recorded in 5‐mm increments (Figure 1).

### 2.5 Radiographical Parameters

**FIGURE 2:**
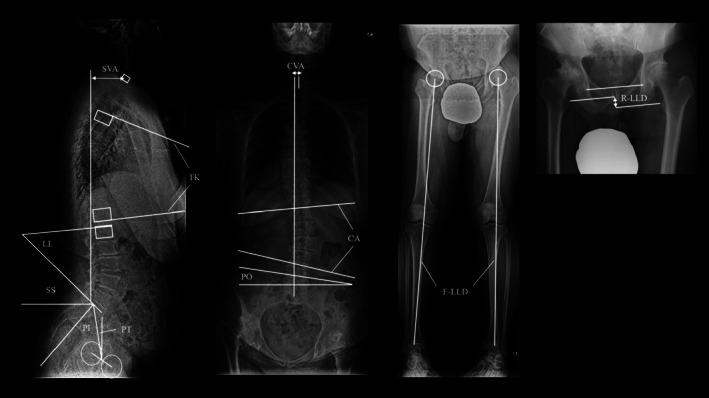
Radiographic parameters. CVA, coronal vertical axis; F‐LLD, functional leg length discrepancy; LL, lumbar lordosis; PI, pelvic incidence; PO, pelvic obliquity; PT, pelvic tilt; R‐LLD, radiographic leg length discrepancy; SS, sacral slope; SVA, sagittal vertical axis; TK, thoracic kyphosis.

Radiographic parameters assessed included the Crowe classification [16], sagittal vertical axis (SVA), thoracic kyphosis (TK), lumbar lordosis (LL), sacral slope (SS), pelvic incidence (PI), pelvic tilt (PT), coronal vertical axis (CVA), Cobb angle, and pelvic obliquity (PO) (Figure 2). Additionally, radiographic leg length discrepancy (R‐LLD) and functional leg length discrepancy (F‐LLD) were evaluated (Figure 2) [11, 17].

The SVA was defined as the distance from the posterior sacral endplate to a perpendicular line passing through the center of the C7 vertebral body. TK was defined as the angle between the line through the superior endplate of T4 and the line through the inferior endplate of T12 [18]. LL was defined as the angle between the superior endplate of L1 and the superior endplate of S1 (line S). SS was defined as the angle between line S and the horizontal line. PT was defined as the angle between the line from the midpoint of the S1 endplate to the acetabular center midpoint (line M) and the vertical line. PI was defined as the angle between line M and a line perpendicular to line S. CVA was defined as the distance between the center of the sacrum and a vertical line descending from the center of C7. PO was defined as the angle between the line connecting the superior margins of both iliac crests and the horizontal line. R‐LLD was measured using the method described by Woolson and Harris [19], while F‐LLD was defined as the difference in length between the center of the hip joint and the center of the ankle joint [20]. The Cobb angle of the lumbar spine was also measured. Forward deviation was defined as positive for SVA, and Cobb angle, CVA, PO, R‐LLD, and F‐LLD were evaluated using absolute values.

To assess interobserver reliability, two orthopedic surgeons independently evaluated 30 randomly selected cases. Both evaluators were blinded to each other's measurements. Intraclass correlation coefficients (ICCs) for each parameter were as follows: SVA: 0.81, TK: 0.78, LL: 0.82, SS: 0.85, PT: 0.74, PI: 0.75, CVA: 0.82, Cobb angle: 0.81, PO: 0.85, R‐LLD: 0.82, and F‐LLD: 0.78.

### 2.6 Data Analysis

The normality of continuous variables was assessed using the Shapiro–Wilk test. For variables that satisfied the assumption of normality, a paired *t*‐test was used to compare pre‐ and postoperative fall risk assessments, and Student's *t*‐test was used for comparisons between groups. Pearson's chi‐square test was applied to categorical variables. When normality was not satisfied, the Wilcoxon signed‐rank test was used. Logistic regression analysis was performed to identify factors associated with fall risk after THA. Statistical significance was set at *p* < 0.05. All statistical analyses were performed using EZR (Version 1.55, Tochigi, Japan) [21].

## 3 Result

### 3.1 Changes in Fall Risk After THA

Total hip arthroplasty significantly reduced the FRI‐5 score from a preoperative mean of 7.79 (6.0–13.0) to 4.56 (0–13.0) postoperatively (*p* < 0.001). In the year before surgery, 36 patients (42.4%) experienced falls, whereas 18 patients (21.2%) experienced falls during the year following surgery, indicating a significant reduction in postoperative falls (*p* = 0.005). Other components of the FRI‐5 also improved after surgery (Table 2).

**TABLE 2:** Difference of FRI‐5 before and after THA.

		Before THA	After THA	*p*
		*n* = 85	*n* = 85	
FRI‐5 score ± SD		7.79 (6.0–13.0)	4.56 (0–13.0)	< 0.001
History of fall	*n* (%)	36 (42.4)	18 (21.2)	0.005
Rounded back	*n* (%)	57 (67.1)	32 (37.6)	< 0.001
Using cane	*n* (%)	58 (68.2)	37 (44.0)	0.002
Decreased walking speed	*n* (%)	81 (95.3)	47 (55.3)	< 0.001
More than five medicines	*n* (%)	45 (52.9)	33 (38.8)	0.09

Abbreviations: FRI‐5, fall risk index 5 items version; THA, total hip arthroplasty.

### 3.2 Comparison Between High‐ and Low‐Risk Groups

At 1 year after THA, 33 patients had an FRI‐5 score of ≥ 6 (high‐risk group), and 52 patients had a score of ≤ 5 (low‐risk group). Compared with preoperative values, 61% of patients demonstrated an improvement in fall risk after THA. The postoperative FRI‐5 score was significantly higher in the high‐risk group (7.75 [6.0–13.0]) than in the low‐risk group (2.44 [0–5.0]) (*p* < 0.001). Preoperatively, the FRI‐5 score did not differ significantly between the two groups (8.31 [6.0–13.0] vs. 7.44 [6.0–13.0], *p* = 0.063).

### 3.3 Patient Characteristics

A significant age difference was observed between the high‐risk and low‐risk groups, with mean ages of 69.1 years (41.0–84.0) and 63.0 years (40.0–86.0), respectively (*p* = 0.006). The proportions of women (93.9% vs. 94.2%) and BMI values (24.2 kg/m^2^ [17.1–33.7] vs. 23.9 kg/m^2^ [16.5–34.0]) were similar between groups. Before surgery, contralateral HOA was present in 13 (39.4%) patients in the high‐risk group and 18 (34.6%) in the low‐risk group; after surgery, the values were 9 (27.3%) and 8 (15.4%), respectively, with no significant differences at either time point (Table 3).

**TABLE 3:** Comparison between two groups based on risk of falls after surgery.

			High‐risk		Low‐risk		*p*
			*n* = 33		*n* = 52		
Age (years)	Median (range)		69.1	(41.0–84.0)	63.0	(40.0–86.0)	0.006
Sex	*n* (%)	Women	31	(93.9)	49	(94.2)	1
		Men	2	(6.1)	3	(5.8)	
BMI (kg/m^2^)	Median (range)		24.2	(17.1–33.7)	23.9	(16.5–34.0)	0.794
FRI‐5 score							
Before THA	Median (range)		8.31	(6.0–13.0)	7.44	(6.0–13.0)	0.063
After THA	Median (range)		7.75	(6.0–13.0)	2.44	(0–5.0)	< 0.001
Other side							
Before THA	*n* (%)	Normal	15	(45.5)	30	(57.7)	0.401
		OA	13	(39.4)	18	(34.6)	
		THA	5	(15.2)	4	(7.7)	
After THA	*n* (%)	Normal	15	(45.5)	30	(57.7)	0.266
		OA	9	(27.3)	8	(15.4)	
		THA	9	(27.3)	14	(26.9)	
Bilateral THA	*n* (%)		4	(12.5)	10	(20.8)	0.385
Crowe classification	*n* (%)	1	26	(78.8)	41	(78.8)	0.721
		2	5	(15.2)	8	(15.4)	
		3	1	(3.0)	3	(5.8)	
		4	1	(3.0)	0	(0)	
HHS	Median (range)	Before	58.6	(38.0–85.0)	56.1	(31.0–78.0)	0.424
		After	86.9	(68.0–100.0)	89.9	(65.0–100)	0.168
P‐LLD	(cm) ±SD	Before	1.23	(0–3.0)	1.30	(0–2.0)	0.823
		After	0.76	(0–3.0)	0.28	(0–2.0)	0.005

Abbreviations: BMI: body mass index; HHS, Harris hip score; JHEQ, Japanese Orthopedic Association Hip‐Disease Evaluation Questionnaire; OA, osteoarthritis; P‐LLD, perceived leg length discrepancy; THA, total hip arthroplasty.

### 3.4 Hip Function and Leg Length Discrepancy

Regarding hip function, the preoperative HHS was 58.6 (38.0–85.0) in the high‐risk group and 56.1 (31.0–78.0) in the low‐risk group. Postoperatively, HHS improved to 86.9 (68.0–100.0) and 89.9 (65.0–100.0), respectively, with no significant difference between the groups. In contrast, postoperative P‐LLD was significantly larger in the high‐risk group than in the low‐risk group (0.71 cm [0–3.0] vs. 0.23 cm [0–2.0], *p* = 0.001).

### 3.5 Radiographic Parameters

Regarding radiographic parameters, no significant difference was observed in the Crowe classification. However, preoperative SVA was significantly larger in the high‐risk group than in the low‐risk group (59.9 mm [0–129.8] vs. 36.9 mm [−42.9–147.2], *p* = 0.039). Postoperative SVA remained significantly larger in the high‐risk group (56.8 mm [0–172.0] vs. 30.9 mm [−54.2–128.8], *p* = 0.034). Preoperative LL was significantly lower in the high‐risk group (32.7° [1.39–79.0]) than in the low‐risk group (41.7° [10.9–63.2]) (*p* = 0.021). No significant differences were found in the remaining radiographic parameters (Table 4).

**TABLE 4:** Radiographical parameter.

THA			High‐risk		Low‐risk		*p*
			*n* = 33		*n* = 52		
			Mean	Range	Mean	Range	
Before	Cobb	(°)	9.9	0–24.8	9.9	0–44.5	0.997
	CVA	(mm)	23.1	2.93–94.4	14.3	0–54.4	0.071
	PO	(°)	2.5	0–9.8	2.3	0–12.2	0.758
	R‐LLD	(mm)	10	0.18–37.6	12.4	0.8–61.7	0.407
	F‐LLD	(mm)	5.3	0–19.0	9.8	0–57	0.069
	SVA	(mm)	59.9	0–129.8	36.9	−42.9–147.2	0.039
	TK	(°)	30.1	0.72–61.8	36	10.8–61.3	0.073
	LL	(°)	32.7	1.39–79	41.7	10.9–63.2	0.021
	SS	(°)	31.6	8.93–66	35.9	18.2–47.1	0.134
	PI	(°)	49.5	28.3–78.9	53.2	29.9–86	0.276
	PT	(°)	19.3	0.8–67.5	18.1	−1.6–52.2	0.731
After	Cobb	(°)	10.9	0–33.0	9.7	0–39.8	0.634
	CVA	(mm)	13.2	0–34.4	14.2	0–36.2	0.701
	PO	(°)	3	0–7.2	2.2	0–10.5	0.237
	R‐LLD	(mm)	8.8	0–11.0	7.2	0–37.4	0.476
	F‐LLD	(mm)	8.8	0–25.0	7.1	0–41.6	0.380
	SVA	(mm)	56.8	0–172.0	30.9	−54.2–128.8	0.034
	TK	(°)	32.2	2.4–64.3	38.1	14.9–60.0	0.096
	LL	(°)	35.9	0.79–69.1	42.2	22.8–66.4	0.160
	SS	(°)	30.1	1.4–56.0	36.4	13.6–50.6	0.069
	PI	(°)	49.1	30.8–80.8	54.4	34.2–80.6	0.124
	PT	(°)	20.2	2.7–62.5	18.5	−3.1–49.7	0.613

Abbreviations: CA, Cobb angle; CVA, coronal vertical axis; F‐LLD, functional leg length discrepancy; LL, lumber lordosis; PI, pelvic incidence; PO, pelvic obliquity; PT, pelvic tilt; R‐LLD, radiographic leg length discrepancy; SS, sacral slope; SVA, sagittal vertical axis; THA, total hip arthroplasty; TK, thoracic kyphosis.

### 3.6 Logistic Regression Analysis

Logistic regression analysis was conducted using age, preoperative SVA, preoperative LL, postoperative SVA, and postoperative P‐LLD as explanatory variables, with postoperative high fall risk as the dependent variable. Factors significantly associated with residual postoperative fall risk were age (OR: 1.20, 95% CI: 1.05–1.36, *p* = 0.006), lower preoperative LL (OR: 0.944, 95% CI: 0.892–0.999, *p* = 0.046), and greater postoperative P‐LLD (OR: 5.81, 95% CI: 1.23–27.5, *p* = 0.026) (Table 5).

**TABLE 5:** Logistic regression.

		OR	95% CI			*p*
Age		1.2	1.05	—	1.36	0.006
SVA	Before THA	0.983	0.954	—	1.01	0.267
LL	Before THA	0.944	0.892	—	0.999	0.046
SVA	After THA	1.02	0.992	—	1.05	0.172
P‐LLD	After THA	5.81	1.23	—	27.5	0.026

Abbreviations: LL, lumber lordosis; P‐LLD, perceived leg length discrepancy; SVA, sagittal vertical axis; THA, total hip arthroplasty.

## 4 Discussion

This study evaluated the risk of falls 1 year after THA in patients with DHOA who were identified as being at risk of falls preoperatively. The results demonstrated that 61% of patients showed an improved fall risk assessment 1 year after THA. Factors that remained significantly associated with postoperative fall risk were advanced age, lower preoperative LL, and greater postoperative P‐LLD.

### 4.1 Effect of THA on Fall Risk in Patients With DHOA

From an anatomical perspective, patients with DHOA tend to exhibit inadequate acetabular coverage, leg‐length discrepancy, and pelvic obliquity, all of which can impair gait stability and increase fall risk [11]. Whether THA increases or decreases the risk of falls remains controversial. Ikutomo et al. reported no reduction in the number of falls after THA [5], and Smith et al. observed that the annual number of falls in post‐THA patients was comparable to that in individuals with lower‐extremity osteoarthritis [7]. However, these previous studies frequently included patients who had no fall risk preoperatively, limiting the ability to detect postoperative improvement. In contrast, our study focused exclusively on patients with DHOA who already exhibited a high preoperative fall risk, and we observed significant improvements both in the number of falls and in fall risk assessment scores 1 year after THA. Therefore, THA may mitigate fall risk specifically in patients with DHOA by correcting biomechanical abnormalities.

### 4.2 Influence of Age and Patient Characteristics on Residual Fall Risk

Aging has been reported in many studies as a factor associated with fall risk. Imagama et al. surveyed a community of older adults and found that age‐related declines in body balance, muscle strength, and physical ability contribute to falls [9]. Additionally, women may experience a higher fall risk due to lower muscle strength compared with men [22]. In this study, aging was identified as a significant risk factor for residual fall risk after THA, indicating that age‐related vulnerability should be considered in older patients undergoing THA. However, no significant difference in sex distribution was observed between the two groups. Although a higher risk of falls has been reported among women with DHOA [11], the limited number of men in our cohort may have influenced the absence of a sex‐related difference [22].

### 4.3 Hip Function, Perceived Leg‐Length Discrepancy, and Fall Risk

Hip function—including pain, impaired mobility, and decreased range of motion—is associated with fall risk [5, 7, 12]. Specifically, low HHS in patients with DHOA has been linked to an increased likelihood of falls [11]. Regarding hip function after THA, Iversen et al. reported that patients with leg‐length discrepancies were more likely to fall [23]. In our study, P‐LLD remained a residual risk factor for falls post‐THA. While DHOA typically presents with significant R‐LLD preoperatively, R‐LLD and P‐LLD do not always correspond. Takemoto et al. reported that 25% of patients perceived an LLD even after radiographic correction following THA [20]. Therefore, the possibility of postoperative P‐LLD should be considered.

### 4.4 Spinopelvic Alignment and Postoperative Stability

THA has been reported to influence spinopelvic balance, although its mechanisms remain unclear [13, 24]. Prior studies have shown that lower LL is common in patients with lumbar spine disorders or persistent low back pain after THA [25, 26], and that Japanese patients tend to have lower LL and greater lumbar mobility than Western populations [27]. Limited lumbar mobility has also been linked to postoperative P‐LLD, which may impair balance [15]. In our study, lower preoperative LL was associated with a higher postoperative fall risk, suggesting that insufficient lumbar compensatory capacity contributes to instability even after THA. Recent meta‐analytic evidence also indicates that pelvic tilt often remains unchanged after pelvic corrective procedures, whereas age and sagittal alignment play dominant roles in postoperative functional stability [24, 28]. These findings highlight the importance of individual spinopelvic parameters—particularly LL—in determining postoperative balance and fall risk.

### 4.5 Strengths and Limitations

This study has several limitations. First, it was a retrospective study with a relatively small sample size, and a post hoc power analysis suggested that the sample may have been insufficient to detect small effect sizes. Second, patients with low preoperative fall risk were excluded, and the effect of THA on those patients remains unclear. Hip function was assessed only with the HHS, which may have a ceiling effect in this population. Third, this study did not evaluate postoperative rehabilitation. Therefore, potential confounding effects related to the intensity of postoperative rehabilitation or physical therapy cannot be excluded. Additionally, the primary outcome was the FRI‐5 score rather than actual fall events because some FRI‐5 components are influenced by pain; postoperative improvement may partially reflect pain relief. To address this, objective measures such as postoperative P‐LLD and spinopelvic parameters were also analyzed, indicating that biomechanical factors contribute to residual fall risk. Despite these limitations, this study is the first to demonstrate that THA may reduce fall risk in patients with DHOA who already have a high preoperative fall risk.

## 5 Conclusion

In conclusion, THA significantly reduced the risk of falls associated with DHOA. Factors that increased the risk of falls were aging, low preoperative LL, and postoperative P‐LLD. These findings highlight the importance of careful preoperative assessment of lumbar spine flexibility and leg‐length balance, as well as surgical strategies aimed at minimizing postoperative P‐LLD.

## Author Contributions

T.A. contributed to writing – review and editing of the manuscript. Y.O. contributed to conceptualization and methodology. Y.T. contributed to methodology. H.F. contributed to validation. Y.O. contributed to validation and investigation. S.I. contributed to supervision of the study. All authors have read and approved the final version of the manuscript and agree to be accountable for all aspects of the work.

## Funding

The authors have nothing to report.

## Conflicts of Interest

The authors declare no conflicts of interest.

## Data Availability

Research data are not shared.

## References

[1] Falls (World Health Organization, 2024), https://www.who.int/news‐room/fact‐sheets/detail/falls.

[2] T. O. Smith , E. Higson , M. Pearson , and M. Mansfield , “Is There an Increased Risk of Falls and Fractures in People With Early Diagnosed Hip and Knee Osteoarthritis? Data From the Osteoarthritis Initiative,” International Journal of Rheumatic Diseases 21, no. 6 (2018): 1193–1201, 10.1111/1756. 27153388

[3] L. Yuan and C. Shih , “Dislocation After Total Hip Arthroplasty,” Archives of Orthopaedic and Trauma Surgery 119, no. 5–6 (1999): 263–266, 10.1007/s004020050406. 10447619

[4] W. M. Ricci , “Periprosthetic Femur Fractures,” Journal of Orthopaedic Trauma 29, no. 3 (2015): 130–137, 10.1097/BOT.0000000000000282. 25699540

[5] H. Ikutomo , K. Nagai , K. Tagomori , N. Miura , N. Nakagawa , and K. Masuhara , “Incidence and Circumstances of Falls in Women Before and After Total Hip Arthroplasty: A Prospective Cohort Study,” Journal of Arthroplasty 33, no. 7 (2018): 2268–2272, 10.1016/j.arth.2018.02.006. 29526333

[6] P. Levinger , E. Wee , S. Margelis , et al., “Pre‐Operative Predictors of Post‐Operative Falls in People Undergoing Total Hip and Knee Replacement Surgery: A Prospective Study,” Archives of Orthopaedic and Trauma Surgery 137, no. 8 (2017): 1025–1033, 10.1007/s00402-017-2727-6. 28597247

[7] T. O. Smith , M. Pearson , and S. K. Latham , “Are People Following Hip and Knee Arthroplasty at Greater Risk of Experiencing a Fall and Fracture? Data From the Osteoarthritis Initiative,” Archives of Orthopaedic and Trauma Surgery 136, no. 6 (2016): 865–872, 10.1007/s00402-016-2445-5. 26994762

[8] Y. Liu , Y. Yang , H. Liu , W. Wu , X. Wu , and T. Wang , “A Systematic Review and Meta‐Analysis of Fall Incidence and Risk Factors in Elderly Patients After Total Joint Arthroplasty,” Medicine 99, no. 50 (2020): e23664, 10.1097/MD.0000000000023664. 33327354 PMC7738153

[9] S. Imagama , Z. Ito , N. Wakao , et al., “Influence of Spinal Sagittal Alignment, Body Balance, Muscle Strength, and Physical Ability on Falling of Middle‐Aged and Elderly Males,” European Spine Journal 22, no. 6 (2013): 1346–1353, 10.1007/s00586-013-2721-9. 23443680 PMC3676567

[10] C. Teck Ng and M. Pin Tan , “Osteoarthritis and Falls in the Older Person,” Age and Ageing 42, no. 5 (2013): 561–566, 10.1093/ageing/aft070. 23864423

[11] T. Asamoto , Y. Osawa , Y. Takegami , M. Okamoto , H. Iida , and S. Imagama , “Fall Risk in Patient With Dysplastic Hip Osteoarthritis,” International Orthopaedics 48, no. 1 (2024): 221–227, 10.1007/s00264-023-05938-z. 37606767

[12] H. Ikutomo , K. Nagai , K. Tagomori , N. Miura , N. Nakagawa , and K. Masuhara , “Gait Abnormality Predicts Falls in Women After Total Hip Arthroplasty,” Journal of Arthroplasty 33, no. 10 (2018): 3215–3219, 10.1016/j.arth.2018.05.044. 29941382

[13] D. Jain , J. M. Vigdorchik , E. Abotsi , et al., “The Impact of Global Spinal Alignment on Standing Spinopelvic Alignment Change After Total Hip Arthroplasty,” Global Spine Journal 13, no. 5 (2023): 1252–1256, 10.1177/21925682211026633. 34142571 PMC10416580

[14] J. Okochi , K. Toba , T. Takahashi , et al., “Simple Screening Test for Risk of Falls in the Elderly,” Geriatrics & Gerontology International 6, no. 4 (2006): 223–227, 10.1111/j.1447-0594.2006.00352.x.

[15] D. Koga , T. Jinno , A. Okawa , S. Morita , and K. Shinomiya , “The Effect of Preoperative Lateral Flexibility of the Lumbar Spine on Perceived Leg Length Discrepancy After Total Hip Arthroplasty,” Journal of Medical and Dental Sciences 56 (2009): 69–77. 19697521

[16] P. K. Wu and Y. M. Lin , “In Brief: Crowe's Classification: Arthroplasty in Developmental Dysplasia of the Hip,” Clinical Orthopaedics and Related Research 468, no. 12 (2010): 3426–3427, 10.1007/s11999-010-1599-7. 20878556 PMC2974878

[17] G. Takemoto , Y. Osawa , T. Seki , et al., “Factors Influencing Inconsistent Leg Length Discrepancy in Dysplastic Hip Osteoarthritis: A Retrospective Study,” BMC Musculoskeletal Disorders 23, no. 1 (2022): 381, 10.1186/s12891-022-05348-z. 35461275 PMC9034481

[18] J. Ouchida , H. Nakashima , T. Kanemura , et al., “Impact of the Hip Joint Mobility on Whole‐Body Sagittal Alignment: Prospective Analysis in Case With Hip Arthroplasty,” European Spine Journal 31, no. 9 (2022): 2399–2407, 10.1007/s00586-022-07251-6. 35776178

[19] S. T. Woolson and W. H. Harris , “A Method of Intraoperative Limb Length Measurement in Total Hip Arthroplasty,” Clinical Orthopaedics and Related Research 194 (1985): 207–210. 3978918

[20] G. Takemoto , Y. Osawa , T. Seki , et al., “A Large Preoperative Pelvic Oblique Angle Affects Perception of Leg Length Discrepancy After Total Hip Arthroplasty,” Journal of Orthopaedic Science 29, no. 2 (2024): 566–573, 10.1016/j.jos.2023.01.013. 36841713

[21] Y. Kanda , “Investigation of the Freely Available Easy‐To‐Use Software 'EZR' for Medical Statistics,” Bone Marrow Transplantation 48, no. 3 (2013): 452–458, 10.1038/bmt.2012.244. 23208313 PMC3590441

[22] S. Muraki , T. Akune , H. Oka , et al., “Physical Performance, Bone and Joint Diseases, and Incidence of Falls in Japanese Men and Women: A Longitudinal Cohort Study,” Osteoporosis International 24, no. 2 (2013): 459–466, 10.1007/s00198-012-1967-0. 22434204

[23] M. D. Iversen , N. Chudasama , E. Losina , and J. N. Katz , “Influence of Self‐Reported Limb Length Discrepancy on Function and Satisfaction 6 Years After Total Hip Replacement,” Journal of Geriatric Physical Therapy 34, no. 3 (2011): 148–152, 10.1519/JPT.0b013e31820e16dc. 21937905 PMC3179609

[24] Q. Karisch , M. Haertlé , J. Stamp , N. Ramadanov , H. Windhagen , and S. S. Ahmad , “Patients With Borderline Hip Dysplasia Present With Inferior Patient‐Reported Outcomes Compared to True Hip Dysplasia,” Journal of Experimental Orthopaedics 12, no. 3 (2025): e70407, 10.1002/jeo2.70407. 40919491 PMC12409825

[25] Y. Okuzu , K. Goto , Y. Kuroda , T. Kawai , and S. Matsuda , “Preoperative Factors Associated With Low Back Pain Improvement After Total Hip Arthroplasty in a Japanese Population,” Journal of Arthroplasty 37, no. 1 (2022): 69–74, 10.1016/j.arth.2021.08.025. 34600782

[26] J. Beck , H. Brisby , A. Baranto , and O. Westin , “Low Lordosis Is a Common Finding in Young Lumbar Disc Herniation Patients,” Journal of Experimental Orthopaedics 7, no. 1 (2020): 38, 10.1186/s40634-020-00253-7. 32476065 PMC7261711

[27] T. Kobayashi , T. Morimoto , T. Yoshihara , M. Sonohata , C. Rivière , and M. Mawatari , “The Significant Relationship Among the Factors of Pelvic Incidence, Standing Lumbar Lordosis, and Lumbar Flexibility in Japanese Patients With Hip Osteoarthritis: A Descriptive Radiographic Study,” Orthopaedics & Traumatology, Surgery & Research 108, no. 2 (2022): 103123, 10.1016/j.otsr.2021.103123. 34700058

[28] M. Kanto , K. Maruo , T. Tachibana , et al., “Influence of Spinopelvic Alignment on Pelvic Tilt After Total Hip Arthroplasty,” Orthopaedic Surgery 11, no. 3 (2019): 438–442, 10.1111/os.12469. 31148364 PMC6595105

